# A Straightforward but Not Piecewise Relationship between Age and Lymph Node Status in Chinese Breast Cancer Patients

**DOI:** 10.1371/journal.pone.0011035

**Published:** 2010-06-09

**Authors:** Ke-Da Yu, Jun-Jie Li, Gen-Hong Di, Jiong Wu, Zhen-Zhou Shen, Zhi-Ming Shao

**Affiliations:** 1 Department of Breast Surgery, Cancer Center and Cancer Institute, Fudan University, Shanghai, People's Republic of China; 2 Department of Oncology, Shanghai Medical College, Fudan University, Shanghai, People's Republic of China; City of Hope National Medical Center, United States of America

## Abstract

**Purpose:**

To investigate the relationship between age and axillary lymph node (LN) involvement in Chinese breast cancer patients, and to replicate a recently identified piecewise relationship between age and LN involvement.

**Methods:**

A dataset, consisting of 3,715 patients (with complete information on study variables) with operable breast cancer consecutively surgically treated between 1996 and 2006, was derived from the database of Shanghai Cancer Hospital. Univariate and multivariate logistic regression were employed to analyze the relationship between age and LN. We subsequently performed a similar analysis on another dataset including 1,832 consecutive patients treated between 2007 and 2008 to replicate our findings in the first dataset.

**Results:**

A U-shaped relationship (previously observed in two European populations) between age and LN status failed to be replicated in our dataset of Chinese patients. Instead, we observed a linear rather than piecewise relationship. After multivariate adjustment, the linear relationship was still present. Moreover, the interaction between age and LN involvement was not modified by tumor size. The odds of LN involvement decreased by 1.5% for each year increase in age (OR 0.985, 95% CI 0.979–0.991, P<0.001). Breast cancer subtypes were also associated with LN status. Proportions of basal-like and ERBB2+ subtypes decreased with increasing age. The observations in the first dataset were successfully replicated in a second independent dataset.

**Conclusion:**

We confirmed a straightforward but not piecewise relationship between age and LN status in Chinese patients. The different pattern between Chinese and European elderly patients should be considered when making clinical decisions.

## Introduction

Despite relatively lower incidence of breast cancer in Asian countries than in the west [1], China has documented a 20–30% increase in urban areas over the past decade [2], [3]. The disease status of axillary lymph nodes (LNs) is the most significant prognostic factor for patients with breast cancer [4] [5]. At present, although the use of sentinel LNs identification and sampling procedure could be reliably performed in selected early stage patients by a trained multidisciplinary team [6], axillary LN dissection (ALND) is still the mainstay of the surgical management of breast cancer in China [3].

The determination of factors associated with axillary LN involvement may help us predict or identify a subgroup of patients with a low rate of node involvement. Regarding the relationship between age and LN status, some investigator suggested the older patients were associated with an increased probability of nodes involvement [7], while others proposed that tumors of elderly patients were biologically more favorable [8] and had a decrease in LN involvement [9]. In addition, Wildiers et al. recently reported a large sample size study (referred to as the Leuven study) examining the relationship between patient age and risk of axillary LN involvement [10]. Interestingly, they identified a piecewise effect of age on LN positivity. Because of these discordant results, we here decided to perform a large scale retrospective analysis for two aims. One aim was to validate the piecewise U-shaped relationship between age and LN status, which had been replicated in two European patient populations; the other was to explore the relationship between age and LN status in Chinese breast cancer patients, which has been seldom studied.

## Methods

### Study subjects and variables

**Figure 1:**
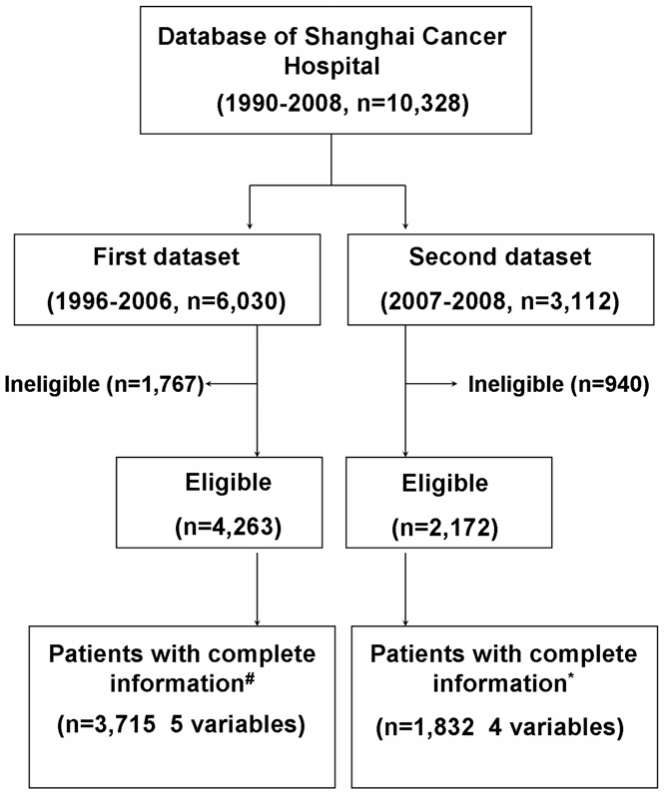
Patient selection and patient subsets analyzed. # 5 variables denote lymph node, age, pathology, tumor size, and subtype * 4 variables denote lymph node, age, pathology, and tumor size.

The study subjects were from the Breast Malignancy Database established by the Department of Surgery, Shanghai Cancer Hospital of Fudan University in Shanghai, China. The database has more than 10,000 case records including detailed clinicopathologic data and follow-up results. The information of this database has been reported elsewhere [3]. All patients gave written informed consent for their information to be stored in the hospital database and used for research, and this study was approved by the Ethical Committee of Shanghai Cancer Hospital of Fudan University. The first dataset we used was derived from the entire database of the period between 1996 and 2006, in which period there were total 6,030 consecutive patients with breast malignancies. Patients selected for the present retrospective study fulfilled the following inclusion criteria: (1) female patients diagnosed with ipsilateral invasive breast cancer. Patients with breast carcinoma *in situ* (with or without microinvasion) and breast sarcoma were excluded; (2) all these patients received primary surgery in our hospital; (3) the pathological examination of patients' tumor specimens was performed in the Department of Pathology in our hospital; (4) patients were operable without any evidence of metastases at diagnosis. Preoperative evaluation and examination had been described previously [11]; (5) patients receiving neoadjuvant systemic therapy (chemotherapy and/or hormone therapy) and preoperative irradiation were excluded. A preoperative diagnostic biopsy was allowed. As a result, 4,263 patients met all these criteria (see **Figure 1** for patient selection).

In order to validate the results of the Leuven study, we chose the following variables for analysis: age at diagnosis (year), pathological tumor size (the largest diameter), tumor grade, pathology (invasive ductal cancer vs. invasive lobular cancer vs. other invasive cancers), involvement of axillary LN (number of positive nodes), estrogen receptor (ER) and progesterone receptor (PR) status, and HER2/neu status. Age at diagnosis and tumor sizes were recorded as continuous variables, the other variables treated as categorical data. Axillary LN status was analyzed either as a binary variable (positive or negative) or as a ternary variable according to the number of involved LNs (0, 1–3, or ≥4). Determination of tumor grading, ER, PR and HER2 status was performed by pathologists in the Department of Pathology in our hospital. The work was done according to established procedures which had been described elsewhere [11].

According to molecular profile of breast cancer subtypes [12], [13], the immunohistochemical (IHC) surrogates for four major subtypes had been developed: luminal A (ER+ and/or PR+, HER2−), luminal B (ER+ and/or PR+, HER2+), ERBB2+ (ER−, PR−, HER2+), and basal-like (ER−, PR−, HER2-, also known as triple-negative) [14]. In the present study, we analyzed the status of ER, PR and HER2 receptors in a combined manner. The luminal A and luminal B breast cancer were combined as the ‘luminal-like subtype’ (ER+ and/or PR+, any HER2 status).

Of the 4,263 eligible breast cancer patients, some of them had missing values of the studied variables, 4.3% of tumor size (n = 182), 12.5% of combined receptors status (n = 532), and 1.4% of axillary LN status (n = 58). Of note, 35.7% cases (n = 1,523) were not evaluated for tumor histological grade (most breast carcinomas of special histologic types such as mucinous, tubular, medullary, and papillary carcinoma were not evaluated). Because of such a high proportion, we did not exclude those cases with missing values of tumor grade. Finally, 3,715 patients (87.1%) with complete information of age, tumor size, pathology, LN status, and ER, PR and HER2 status were selected for analysis.

### Replication of the observed relationship in Chinese patients

The second dataset for validation was also derived from the entire database, and the patients corresponded to the breast cancer cases of the period between 2007 and 2008 (**Figure 1**). During that period, more than 3,000 consecutive breast malignancy patients received primary surgical treatment in our department. A total 3,112 cases were entered into our database. Among these, 2,172 patients fulfilled the inclusion criteria applied for patient selection in the first dataset.

The IHC data (ER/PR/HER2) in the second dataset is currently being checked and replenished, and only half of patients (n = 997, 45.9%) in the second dataset are available for ER, PR and HER2. Therefore, we excluded the independent variable “breast cancer subtype” from further analysis. Percentages of other missing data on tumor size, tumor grade and LN involvement were 15.0%, 20.4% and 3.0%, respectively. Finally, we selected 1,832 (84.3%) patients with complete information (except tumor grade and subtype) for analysis. Similar to the situation in the first dataset, we performed multivariate logistic regression to predict LN involvement using three independent variables (age, tumor size and pathology).

### Statistical analysis

We regressed on age using nonparametric logistic regression based on smoothing (LOWESS) method [10] to explore the relationship pattern in our study subjects. In descriptive statistics, associations between categorical variables were tested using Pearson's χ^2^ test. The odds ratios (OR) with 95% confidence interval (CI) for relationship between each variable and LN involvement (yes or no) were calculated using logistic regression. We employed multivariate logistic regression to predict LN involvement (method: backward stepwise, likelihood ratio). For the first dataset, the regression model was established based on four basic independent variables (age, tumor size, pathology and subtype). Similarly, for the second dataset, the model was established based on three basic independent variables (age, tumor size and pathology). Breast cancer subtype was chosen for modeling in the first dataset, but not in the second dataset. Hosmer-Lemeshow test was performed to assess goodness-of-fit of model. A P-value less than or equal to 0.05 was considered statistically significant. Statistical analysis was performed using Stata/SE version 10.0 (Stata, College Station, TX) and SPSS Software version 12.0 (SPSS, Chicago, IL, USA).

## Results

The basic characteristics of the patients from the first dataset (1996–2006, n = 3,715) are shown in **Table 1**. Since more than half of breast cancer patients in China are premenopausal and the patients older than 80 years are relatively few [2], we arbitrarily divided the study subjects into five groups according to age: younger than 40 years, 40–49 years, 50–59 years, 60–69 years, and 70 years and older.

**Table 1:** Tumor characteristics and lymph node involvement according to age in the two datasets.

Parameter		Age category (years)					
		<40	40–49	50–59	60–69	≥70	Total
**The first dataset, n**		**329**	**1250**	**1133**	**593**	**347**	**3,715**
Median tumor size, cm		2.3	2.5	2.5	2.7	3.0	2.5
Histology, %	Ductal	89.3	90.0	89.8	85.5	81.3	88.3
	Lobular	3.1	3.7	3.1	4.6	3.2	3.5
	Other	7.7	6.3	7.1	9.9	15.6	8.1
Tumor grade*, %	I	1.0	1.6	1.5	1.7	1.7	1.5
	II	38.8	44.0	51.4	48.4	47.8	46.8
	III	23.2	19.3	17.9	17.0	12.7	18.3
	Missing	37.0	35.1	29.2	32.9	37.8	33.4
Involved lymph nodes, %	Negative	46.2	53.4	52.4	60.5	62.5	54.3
	Positive	53.8	46.6	47.6	39.5	37.5	45.7
	1–3	28.3	29.6	28.9	23.4	23.6	27.7
	≥4	25.5	17.0	18.7	16.0	13.8	18.0
ER, %	+	42.6	47.4	56.8	66.3	66.9	54.6
PR, %	+	43.6	54.6	48.1	52.3	52.7	50.9
HER2, %	+	21.9	23.0	25.5	21.2	16.0	22.7
Subtypes, %	Luminal-like	57.7	65.8	67.3	74.2	77.2	67.8
	ERBB2+	13.0	10.8	10.3	7.4	6.1	9.9
	Basal-like	29.3	23.4	22.3	18.4	16.7	22.3
**The second dataset** & **, n**		**242**	**534**	**637**	**295**	**124**	**1,832**
Median tumor size, cm		2.0	2.0	2.1	2.2	2.0	2.0
Histology, %	Ductal	96.7	94.2	94.0	90.2	83.1	93.1
	Lobular	0.4	2.1	0.8	0.7	2.4	1.2
	Other	2.9	3.7	5.2	9.2	14.5	5.7
Tumor grade*, %	I	2.5	3.7	2.2	2.7	3.2	2.8
	II	54.5	56.9	60.6	61.4	60.5	58.8
	III	37.2	31.5	30.3	27.5	25.8	30.8
	Missing	5.8	7.9	6.9	8.5	10.5	7.5
Involved lymph nodes, %	Negative	49.2	52.8	55.7	58.6	62.9	55.0
	Positive	50.8	47.2	44.3	41.4	37.1	45.0
	1–3	28.5	29.2	26.7	25.1	23.4	27.2
	≥4	22.3	18.0	17.6	16.3	13.7	17.8

*Data of tumor grade are available in 66.6% subjects in the first dataset and 92.5% in the second dataset.

&Immunohistochemical data on ER/PR/HER2 status in the dataset of 2008 is currently being checked and replenished. Only half of values (n = 997, 45.9%) are available. Therefore, the analysis of variable “breast cancer subtype” is not performed in the second dataset.

### A linear relationship between age and LN involvement in univariate analysis

**Figure 2:**
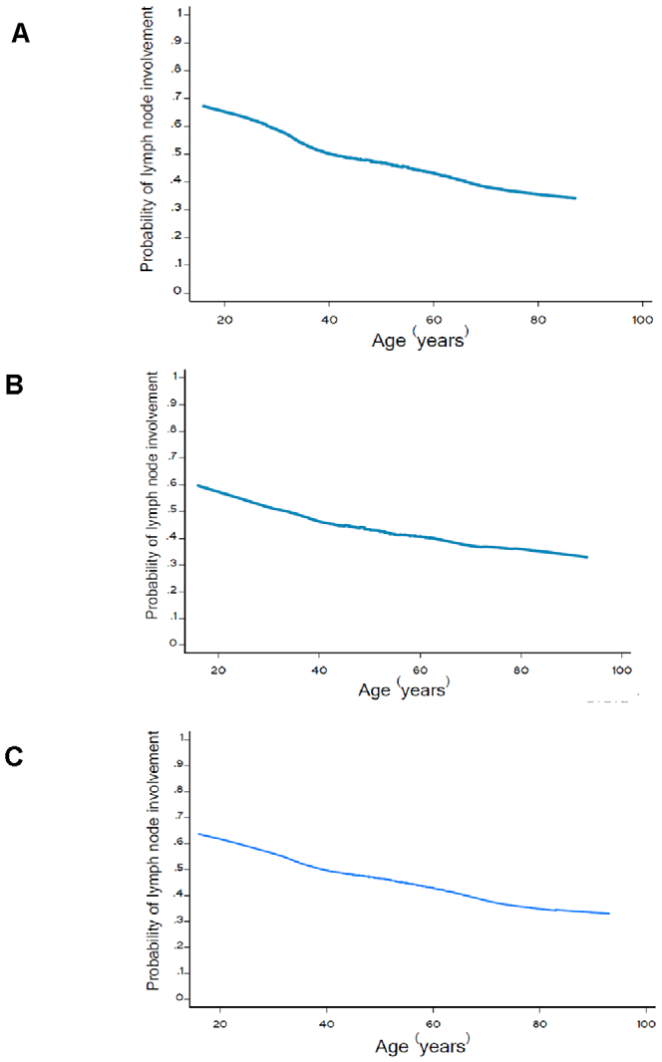
Relation between age and lymph node involvement based on univariate nonparametric smoothing method. (A) The first dataset using univariate nonparametric smoothing method (n = 3,715) (B) The second dataset using univariate nonparametric smoothing method (n = 1,832) (C) The combined two datasets using univariate nonparametric smoothing method (n = 5,547).

As the Leuven study previously demonstrated a U-shaped relationship between age and LN involvement [10], we first tried to replicate that result in our population. However, in our study, the LOWESS smoothing plot displayed a linear rather than a piecewise effect of age on LN status (**Figure 2A**). Age was negatively related to LN involvement in the whole group (OR per year 0.985, 95% CI 0.979–0.990 **Table 2**). Other independent variables including tumor size (OR per centimeter 1.244, 95% CI 1.182–1.310), pathology (OR 0.625, 95% CI 0.551–0.709), ER (OR of positive 0.645, 95% CI 0.566–0.735) and PR status (OR of positive 0.820, 95% CI 0.720–0.933), and HER2 status (OR of positive 1.363, 95% CI 1.167–1.591) had significant relationships with LN involvement. When combining ER, PR and HER2 as variables of breast cancer subtype, ERBB2+ and basal-like subtypes, both contributed to the risk of LN involvement (OR 1.773, 95% CI 1.543–2.037).

**Table 2:** Univariante and multivariate logistic regression for lymph node involvement in the first and the second dataset.

Independent variable	Univariate analysis			Multivariate analysis		
	OR	95% CI	P-value	HR	95% CI	P-value
**The first study (n = 3,715)**						
Age (continuous)	0.985	0.979–0.990	<0.001	0.985	0.979–0.991	<0.001
Size (continuous)	1.244	1.182–1.310	<0.001	1.262	1.197–1.331	<0.001
Pathology (Ductal vs. Lobular vs. Other)	0.625	0.551–0.709	<0.001	0.614	0.540–0.699	<0.001
Subtypes (Luminal-like vs. ERBB+ vs. Basal-like)	1.773	1.543–2.037	<0.001	1.303	1.203–1.411	<0.001
**The second study (n = 1,832)**						
Age (continuous)	0.986	0.978–0.994	0.001	0.989	0.980–0.997	0.009
Size (continuous)	1.409	1.302–1.524	<0.001	1.416	1.309–1.533	<0.001
Pathology (Ductal vs. Lobular vs. Other	0.565	0.449–0.711	<0.001	0.558	0.439–0.710	<0.001

### The repeatable linear relationship between age and LN status

In the second dataset, the univariate effect of age on LN involvement using LOWESS smoothing method also displayed a similar linear relationship that was observed in the first study (**Figure 2B**). Age was negatively related to LN involvement (OR per year 0.986, 95% CI 0.978–0.994 **Table 2**). Other independent variables associated with LN involvement included tumor size (OR per centimeter 1.244, 95% CI 1.182–1.310) and pathology (OR 0.565, 95% CI 0.449–0.711). When we combined the first dataset and the second dataset together, the LOWESS smoothing plot showed the same trend, i.e., that older women had a lower probability of LN involvement (**Figure 2C**).

### Multivariate logistic regression confirmed the linear relationship

After adjustment of other predictors, the linear relationship between age and LN involvement was still present. As the results from the first dataset showed, the odds of LN involvement decreased by 1.5% for each year increase in age (OR 0.985, 95% CI 0.979–0.991), increased by 25–30% for each centimeter increase in tumor size (OR 1.262, 95% CI 1.197–1.331), and increased in ERBB2+ and basal-like subtypes (OR 1.303, 95% CI 1.203–1.411). Special histologic type (such as mucinous, tubular, medullary, and papillary carcinoma) also displayed a decreased risk of LN involvement compared with invasive ductal cancer. Similarly, the reverse relationship between age and LN involvement was successfully replicated.

Since the increase in age had a negative effect on LN involvement, and no piecewise effect was observed, we therefore did not develop piecewise models for prediction of node involvement. One logistic regression model worked well for patients of any age. We regressed a model using four independent variables (age, size, pathology and subtype) from the first dataset and three independent variables (age, size, and pathology) from the second dataset, respectively. The goodness-of-fit tests suggested that both models were good fits. Hazard ratios of each independent variable are presented in **Table 2**. The probability of lymph node involvement was estimated as e^L^/(1+e^L^), where the value of L was derived by multivariate logistic regression analysis. The dependent variable was “lymph node involvement”, and the independent variables for the first dataset model were age, pathology, size, and subtype; the independent variables for the second dataset model were age, pathology, and size. The values of age and size were continuous. For pathology, invasive ductal = 1, invasive lobular = 2, and other invasive carcinomas = 3; for subtype, luminal-like = 1, ERBB+ = 2, and basal-like = 3.

Formula for the first model:
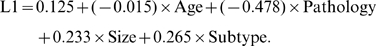



Formula for the second model:
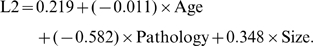



### No modifying effect of tumor size on the relationship between age and LN involvement

**Figure 3:**
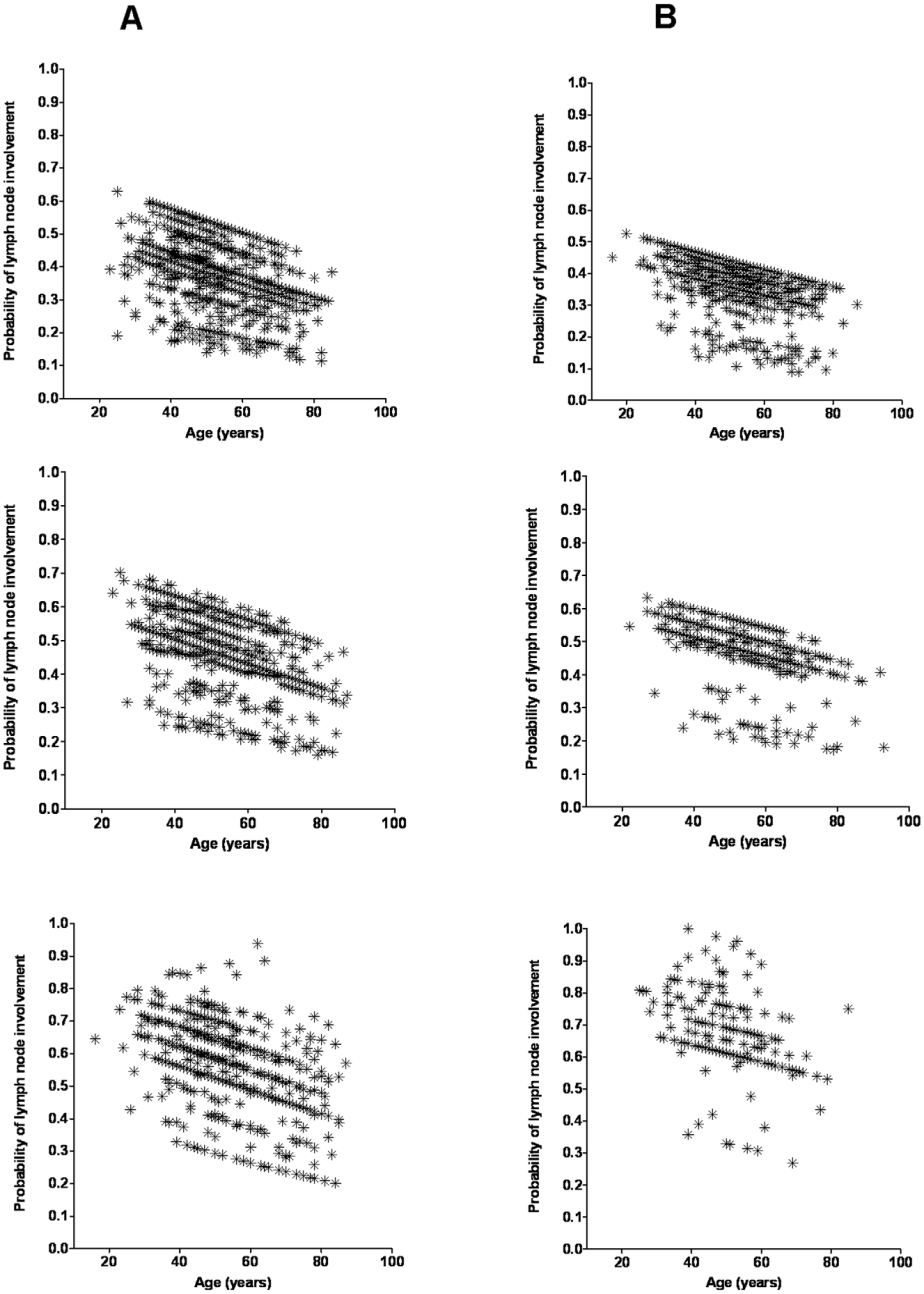
Scatterplots of age and the model's predicted probability of lymph node involvement. These patients have complete data on age at diagnosis, tumor size, axillary lymph node status, and IHC-based subtypes. Separate plots are shown for (**A**) the first dataset (n = 3,715) and (**B**) the second dataset (n = 1,832), and for women with tumor size up to 2.0 cm (top plots), tumor size between 2.1 and 3.5 cm (middle plots), and tumor size greater than 3.5 cm (bottom plots).

The Leuven study revealed an interaction between age and tumor size on the frequency of LN involvement. It was demonstrated that the piecewise effect for age on the LN involvement was clearer in small tumors but not in tumors larger than 3.5 cm [10]. However, in our study, we observed that the effect of age on LN positivity was not affected by tumor size through subgroup analysis (**Table 3**). Despite subdivision of the study subjects according to tumor size (≤2.0 cm, 2.1–3.5 cm, ≥3.6 cm), the reverse relationship between age and LN status did change either in the first dataset or in the combined dataset. Furthermore, the predicted probability of LN involvement was negatively associated with age in each subgroup (**Figure 3A**) in the first dataset, which was replicated in the second dataset (**Figure 3B**).

**Table 3:** Subgroup analysis of the relationship between age and lymph node involvement by tumor size.

Size (cm)	Age (year)	First dataset				Combining two datasets				
		LN-negative		LN-positive		LN-negative		LN-positive		P-value
		n	%	n	%	n	%	n	%	
≤2.0	<40	98	60.9	63	39.1	174	60.6	113	39.4	0.020
	40–49	317	58.5	225	41.5	501	59.8	337	40.2	
	50–59	294	58.3	210	41.7	490	59.6	332	40.4	
	60–69	153	65.1	82	34.9	249	66.2	127	33.8	
	≥70	87	70.2	37	29.8	133	70.0	57	30.0	
2.1–3.5	<40	56	41.2	80	58.8	94	43.1	124	56.9	0.002
	40–49	235	52.9	209	47.1	315	51.6	295	48.4	
	50–59	219	53.9	187	46.1	351	54.3	296	45.7	
	60–69	141	60.8	91	39.2	209	58.2	150	41.8	
	≥70	74	64.3	41	35.7	99	60.0	66	40.0	
≥3.6	<40	27	28.4	68	71.6	32	24.8	97	75.2	<0.0001
	40–49	115	43.6	149	56.4	133	39.6	203	60.4	
	50–59	81	36.3	142	63.7	108	35.9	193	64.1	
	60–69	65	51.6	61	48.4	74	48.4	79	51.6	
	≥70	56	51.9	52	48.1	63	54.3	53	45.7	

## Discussion

A piecewise U-shaped relationship between age and LN involvement has been recently reported for two European breast cancer populations [10]. The present study attempted to validate it in our study subjects of Chinese breast cancer patients. However, our results displayed a linear rather than U-shaped relationship. The linear relationship observed in our first dataset was subsequently replicated in a second independent dataset. Successful validation strengthens the reliability of our results, indicating an increase in age related to a continuous decrease in LN involvement.

There could be a number of reasons responsible for the inconsistence between our study and the Leuven study. First, selection bias may be a causal factor. Results from the National Surgical Adjuvant Breast Project-B04 trial had demonstrated that ALND did not improve survival in patients with clinically negative axillary nodes [15]. Subsequent clinical trials demonstrated that there was no significant survival difference between ALND and no axillary dissection in the selected patients with older age small tumor, and clinically negative axilla LNs [16]–[19]. Therefore, in clinical practice, a certain number of early stage elderly patients received lumpectomy or simple mastectomy only without ALND. These patients were excluded from analysis due to no pathological node status available. As a result, a higher percentage of LN involvement was observed in the older population. This conservative therapeutic strategy seems to be more prevalent in Europe [16]–[19]. In China, however, only a slight proportion of elderly patients were subjected to simple mastectomy or lumpectomy without ALND [3]. The dataset which we used included all the consecutive patients who underwent surgery during the studied period. Every operable elderly patient was included despite her disease stage. In the Leuven study, however, whether all elderly patients during the studied period were recruited is unknown. Second, the epidemiologic difference of breast cancer between the east and the west could influence the outcomes as well. In most European and American countries, the peak of breast cancer incidence was observed in patients >70–75 years old. In contrast, the incidence peak emerged in the middle-age group (45–60 years) in Chinese population [2]. Distinct age distribution of breast cancer patients between the two populations, as well as ethnic heterogeneity between Europeans and Asians, might make the nonrepeatability of the piecewise effect of age on LN involvement.

Our results were in line with the results in other studies. Previous studies suggested that age was negatively associated with node involvement, and tumor of older patients frequently had ER+ phenotype [9], [20]–[23]. Regarding intrinsic subtypes, we found decreasing proportions of basal-like subtype (an aggressive phenotype) and ERBB2+ subtype (another aggressive phenotype) with increasing age. Basal-like breast cancer is associated with higher grade, younger age, higher probability of node involvement and poor prognosis [11], [24], [25]. Luminal-like tumors, however, have a good biologic behavior and are more frequently observed in older patients [20], [23], [26]. Additionally, a special type, mucinous breast carcinoma, is more likely to occur in elderly patients. Mucinous carcinomas have substantially less nodal involvement, have higher expression rates of ER and/or PR, and have a lower S-phase fraction, compared with infiltrating ductal carcinoma [27]. The features mentioned above may determine the indolent nature of breast cancers in elderly women, implying that advancing age is associated with more favorable tumor biology and less involved LNs. Nowadays, sentinel lymph node biopsy (SLNB) has become an alternative procedure of ALND. SLNB presents a lower risk of significant operative morbidity but similar benefit, and has emerged as the milestone advance in the surgical management of early-stage primary breast cancer [28], [29]. In our hospital, the proportion of SLNB in the early breast cancer is relatively low (approximately 10%). Considering the low number of SLNB performed in our hospital and the low false-negative rate (<5%), we did not think SLNB would cause obvious bias of this study.

Some limitations of our study should be acknowledged. First, the first dataset had missing values of tumor grade, and the second dataset included no information on ER/PR/HER2 status. High percentage of missing values could result in increased likelihood of biased results. We predicted the probability of LN involvement using partial independent variables; a stable, robust linear relationship was repeatedly observed in different models. Second, the studied period of the first dataset was fairly long. There should be unavoidable biases in surgical procedure, histopathology evaluation, and ER/PR/HER2 detection. Third, the second dataset for validation, although independent, was also derived from the entire database, which could have increased the probability of successful validation. Fourth, in our Chinese database, the proportion of ≥80 years-old patients was low, and there were only 79 women with age ≥80 years, even after combining these two datasets. The small proportion of elderly and the under-representation of the ≥80 years-old group might explain the lack of reproducibility.

So far, most studies showed a linear rather than piecewise relationship between age and LN involvement. Some observations in the Leuven study are not straightforward to explain. Typically, although the authors offered the possible biologic reasons for their findings, such as the possibility that suppressed cellular immunity in the elderly might offset the favorable tumor biology, this explanation was not consistent with the observation that the increased rate of axillary node involvement in older patients versus younger patients was only limited to patients with smaller tumors [30]. We stress that the novel piecewise relationship should be further verified in other populations.

In conclusion, we confirmed a straightforward but not piecewise relationship between age and LN status in Chinese breast cancer patients. The U-shaped relationship observed in European breast cancer patients does not appear to be applicable to breast cancer patients in China. The ethnic differences in LN involvement in elderly patients should be considered when making clinical decisions, as well as when establishing global clinical practice guidelines.

## References

[1] Jemal A, Siegel R, Ward E, Murray T, Xu J (2006). Cancer statistics, 2006.. CA Cancer J Clin.

[2] Fan L, Zheng Y, Yu KD, Liu GY, Wu J (2009). Breast cancer in a transitional society over 18 years: trends and present status in Shanghai, China.. Breast Cancer Res Treat in press.

[3] Yu KD, Di GH, Wu J, Lu JS, Shen KW (2007). Development and trends of surgical modalities for breast cancer in China: a review of 16-year data.. Ann Surg Oncol.

[4] de Boer M, van Dijck JA, Bult P, Borm GF, Tjan-Heijnen VC (2010). Breast cancer prognosis and occult lymph node metastases, isolated tumor cells, and micrometastases.. J Natl Cancer Inst.

[5] Hogan BV, Peter MB, Shenoy H, Horgan K, Shaaban A (2010). Intramammary lymph node metastasis predicts poorer survival in breast cancer patients.. Surg Oncol.

[6] Rutgers EJ (2008). Sentinel node biopsy: interpretation and management of patients with immunohistochemistry-positive sentinel nodes and those with micrometastases.. J Clin Oncol.

[7] Molino A, Giovannini M, Auriemma A, Fiorio E, Mercanti A (2006). Pathological, biological and clinical characteristics, and surgical management, of elderly women with breast cancer.. Crit Rev Oncol Hematol.

[8] Diab SG, Elledge RM, Clark GM (2000). Tumor characteristics and clinical outcome of elderly women with breast cancer.. J Natl Cancer Inst.

[9] Singh R, Hellman S, Heimann R (2004). The natural history of breast carcinoma in the elderly: implications for screening and treatment.. Cancer.

[10] Wildiers H, Van Calster B, van de Poll-Franse LV, Hendrickx W, Roislien J (2009). Relationship between age and axillary lymph node involvement in women with breast cancer.. J Clin Oncol.

[11] Yin WJ, Lu JS, Di GH, Lin YP, Zhou LH (2009). Clinicopathological features of the triple-negative tumors in Chinese breast cancer patients.. Breast Cancer Res Treat.

[12] Perou CM, Sorlie T, Eisen MB, van de Rijn M, Jeffrey SS (2000). Molecular portraits of human breast tumours.. Nature.

[13] Sorlie T, Perou CM, Tibshirani R, Aas T, Geisler S (2001). Gene expression patterns of breast carcinomas distinguish tumor subclasses with clinical implications.. Proc Natl Acad Sci U S A.

[14] Carey LA, Perou CM, Livasy CA, Dressler LG, Cowan D (2006). Race, breast cancer subtypes, and survival in the Carolina Breast Cancer Study.. Jama.

[15] Fisher B, Jeong JH, Anderson S, Bryant J, Fisher ER (2002). Twenty-five-year follow-up of a randomized trial comparing radical mastectomy, total mastectomy, and total mastectomy followed by irradiation.. N Engl J Med.

[16] Veronesi U, Orecchia R, Zurrida S, Galimberti V, Luini A (2005). Avoiding axillary dissection in breast cancer surgery: a randomized trial to assess the role of axillary radiotherapy.. Ann Oncol.

[17] Martelli G, Boracchi P, De Palo M, Pilotti S, Oriana S (2005). A randomized trial comparing axillary dissection to no axillary dissection in older patients with T1N0 breast cancer: results after 5 years of follow-up.. Ann Surg.

[18] Louis-Sylvestre C, Clough K, Asselain B, Vilcoq JR, Salmon RJ (2004). Axillary treatment in conservative management of operable breast cancer: dissection or radiotherapy? Results of a randomized study with 15 years of follow-up.. J Clin Oncol.

[19] Martelli G, Miceli R, De Palo G, Coradini D, Salvadori B (2003). Is axillary lymph node dissection necessary in elderly patients with breast carcinoma who have a clinically uninvolved axilla?. Cancer.

[20] Ma CD, Zhou Q, Nie XQ, Liu GY, Di GH (2009). Breast cancer in Chinese elderly women: pathological and clinical characteristics and factors influencing treatment patterns.. Crit Rev Oncol Hematol.

[21] Yu KD, Di GH, Wu J, Lu JS, Shen KW (2008). Breast cancer patients with estrogen receptor-negative/progesterone receptor-positive tumors: being younger and getting less benefit from adjuvant tamoxifen treatment.. J Cancer Res Clin Oncol.

[22] Yu KD, Liu GY, Di GH, Wu J, Lu JS (2007). Progesterone receptor status provides predictive value for adjuvant endocrine therapy in older estrogen receptor-positive breast cancer patients.. Breast.

[23] Crivellari D, Aapro M, Leonard R, von Minckwitz G, Brain E (2007). Breast cancer in the elderly.. J Clin Oncol.

[24] Rakha EA, El-Sayed ME, Reis-Filho J, Ellis IO (2009). Patho-biological aspects of basal-like breast cancer.. Breast Cancer Res Treat.

[25] Lee JH, Kim SH, Suh YJ, Shim BY, Kim HK (2010). Predictors of Axillary Lymph Node Metastases (ALNM) in a Korean Population with T1-2 Breast Carcinoma: Triple Negative Breast Cancer has a High Incidence of ALNM Irrespective of the Tumor Size.. Cancer Res Treat.

[26] Bertucci F, Finetti P, Cervera N, Charafe-Jauffret E, Buttarelli M (2009). How different are luminal A and basal breast cancers?. Int J Cancer.

[27] Diab SG, Clark GM, Osborne CK, Libby A, Allred DC (1999). Tumor characteristics and clinical outcome of tubular and mucinous breast carcinomas.. J Clin Oncol.

[28] Takei H, Kurosumi M, Yoshida T, Ishikawa Y, Hayashi Y (2010). Axillary lymph node dissection can be avoided in women with breast cancer with intraoperative, false-negative sentinel lymph node biopsies.. Breast Cancer.

[29] Giuliano AE, Chung AP (2010). Long-term follow-up confirms the oncologic safety of sentinel node biopsy without axillary dissection in node-negative breast cancer patients.. Ann Surg.

[30] Mamounas EP (2009). Age and lymph node status in breast cancer: not a straightforward relationship.. J Clin Oncol.

